# Labor Market Integration of People with Disabilities: Results from the Swiss Spinal Cord Injury Cohort Study

**DOI:** 10.1371/journal.pone.0166955

**Published:** 2016-11-22

**Authors:** Jan D. Reinhardt, Marcel W. M. Post, Christine Fekete, Bruno Trezzini, Martin W. G. Brinkhof, on behalf of SwiSCI Study Group

**Affiliations:** 1 Swiss Paraplegic Research, Nottwil, Switzerland; 2 University of Lucerne, Department of Health Sciences and Health Policy, Lucerne, Switzerland; 3 Institute for Disaster Management and Reconstruction, Sichuan University and Hong Kong Polytechnic University, Chengdu, China; 4 Center of Excellence in Rehabilitation Medicine, Brain Center Rudolf Magnus, University Medical Center and De Hoogstraat Rehabilitation, Utrecht, the Netherlands; 5 University of Groningen, University Medical Center Groningen, Department of Rehabilitation Medicine, Groningen, the Netherlands; Universita degli Studi di Perugia, ITALY

## Abstract

**Objectives:**

We aimed to describe labor market participation (LMP) of persons with spinal cord injury (SCI) in Switzerland, to examine potential determinants of LMP, and to compare LMP between SCI and the general population.

**Methods:**

We analyzed data from 1458 participants of employable age from the cross-sectional community survey of the Swiss Spinal Cord Injury Cohort Study. Data on LMP of the Swiss general population were obtained from the Swiss Federal Statistical Office. Factors associated with employment status as well as the amount of work performed in terms of full-time equivalent (FTE) were examined with regression techniques.

**Results:**

53.4% of the participants were employed at the time of the study. Adjusted odds of being employed were increased for males (OR = 1.73, 95% CI 1.33–2.25) and participants with paraplegia (OR = 1.78, 95% CI 1.40–2.27). The likelihood of being employed showed a significant concave relationship with age, peaking at age 40. The relation of LMP with education was s-shaped, while LMP was linearly related to time since injury. On average, employment rates were 30% lower than in the general population. Males with tetraplegia aged between 40 and 54 showed the greatest difference. From the 771 employed persons, the majority (81.7%) worked part-time with a median of 50% FTE (IRQ: 40%-80%). Men, those with younger age, higher education, incomplete lesions, and non-traumatic etiology showed significantly increased odds of working more hours per week. Significantly more people worked part-time than in the general population with the greatest difference found for males with tetraplegia aged between 40 and 54.

**Conclusions:**

LMP of persons with SCI is comparatively high in Switzerland. LMP after SCI is, however, considerably lower than in the general population. Future research needs to show whether the reduced LMP in SCI reflects individual capacity adjustment, contextual constraints on higher LMP or both.

## Introduction

Across the world, persons with disabilities are less likely to be employed or to receive proper assistance and education for employment than the general population [1–3]. This situation entails enormous economic and psychosocial costs, first for the individual in terms of poverty, decreased self-esteem, impaired health, and worse overall social integration and participation [4, 5] and second, for the society in terms of increased social and health care spending, loss of tax revenue and loss of resources such as diversity and creativity [2, 5]. Moreover, work and employment fulfill important social functions for people with disabilities including recognition as full members of society, obtaining income, establishing contacts with other people, contributing to society, and structuring time [6, 7].

Considerable variation regarding labor market participation (LMP) of persons with disabilities has been observed, even across highly developed economies [2, 8]. Against this background, Switzerland provides an interesting example as a high resource-country with low unemployment rates of the general population and an increasing focus of social policies on reintegration of persons with disabilities into the labor market in the last decades [9].

To study LMP of persons with disabilities in-depth, spinal cord injury (SCI) may serve as a case in point. SCI is a major health condition affecting all bodily functions below the lesion level, and may have a substantial impact on activities and social participation against the background of environmental barriers such as inaccessible infrastructure and negative attitudes [10–12]. While SCI is a severe health condition, it usually does not entail major cognitive impairments. Consequently, prospects for employment are good, if sufficient support and accommodations are made available [13]. Employment rates of persons with SCI are nonetheless well below those of the general population [5, 13, 14].

Employment rates and predictors for return to work of people with SCI have been studied extensively over the past decades [4, 5, 13–17]. However, there are several shortcomings in current research. First, most studies rely on highly selective samples or investigate return to work of persons who were employed at time of SCI onset, thus potentially introducing selection bias. Second, past investigations of determinants of LMP after SCI did not consider potential non-linear associations of determinants such as age, time since injury, or years of formal education with the outcome, whereas the likelihood of being employed may, for example, increase up to a certain age and then decrease again. Third, most of the available studies use LMP as binary outcome (employed vs. non-employed) and do not include more detailed information about the amount of work performed (e.g. hours per week). Fourth, comparisons of LMP in SCI with age- and gender-matched general population samples are scarce. However, such comparison is essential in order to account for the overall performance of the labor market of a particular country.

The only study which assessed LMP in persons with SCI in Switzerland before [18] suffered from limited generalizability of its findings since the sampling frame was restricted to a single organization for persons with disabilities (Swiss Paraplegic Association, SPA) and the response rate was low (27%).

By using data from the largest community survey on person with SCI in Europe, we have the opportunity to address the before mentioned shortcomings of current research on LMP in SCI. More specifically, the population-based community survey of the Swiss Spinal Cord Injury Cohort Study (SwiSCI) provides data from almost 2000 persons with SCI and thus enables us to provide more accurate and less biased estimates relying on a large sample of the SCI population in Switzerland [19, 20]. Moreover, we investigate non-linear relationships with selected determinants and consider the amount of work performed, in terms of full-time equivalent (FTE), in our analysis. Finally, by using an age- and gender-matched sample of the Swiss general population, we are able to illustrate differences in LMP between persons with SCI and the general population.

The aims of this study are 1) to report employment rates of people with SCI living in Switzerland and to examine their variation by sociodemographic and lesion characteristics, 2) to compare employment rates between the SCI and the general population providing age- and gender-matched differences, 3) to analyze the amount of work performed by those employed and its association with sociodemographic and lesion characteristics, and 4) to provide a comparison between FTE of people with SCI and the general population.

## Materials and Methods

### Design

The study draws on cross-sectional data from the first SwiSCI community survey [19–21] performed between late 2011 and early 2013. The community survey was conducted according to the ethical principles formulated in the Declaration of Helsinki and its study protocol was approved by the responsible Medical Ethics Committee of the Canton of Lucerne and subsequently by the Ethics Committees of all other involved Swiss cantons, namely Zurich, Basel, and Valais. The legal age of consent in Switzerland is 16 years and written informed consent for participation in the study was obtained from all subjects prior to data collection.

Data were collected using a mixed-mode approach offering participants the opportunity to respond by postal or online questionnaire. Also, telephone interviews were optional in special cases (e.g., for persons with limited hand function). Following a written invitation, a proactive reminder management including up to two written reminders and one telephone reminder was implemented to improve response rates and minimize response bias. Data were stored in a central database and anonymized before analysis. Further details on the study protocol, the recruitment procedures and the reminder management can be found elsewhere [20, 21].

### Sample

Eligible subjects were Swiss residents with a traumatic or non-traumatic SCI aged 16 years or older and living in the community. Persons with congenital conditions leading to SCI, with new SCI in the context of palliative care, with neurodegenerative disorders, and with Guillain-Barré syndrome were excluded from the study. Potential participants were identified from databases of the SPA, three SCI clinical centers, and an SCI home-care service provider (ParaHelp). This approach was chosen since a central registry covering all persons with SCI in Switzerland does not exist to date. As a consequence, the sampling frame did not include all elements of the target population, i.e. all persons aged 16 years or older living with SCI in Switzerland, but the subgroup of those being a member of the SPA, having been treated in one of the above mentioned centers, or being a client of the home-care service provider ParaHelp. In total, 3144 persons were eligible for the study of which 1922 participated in the survey, indicating a response rate of 61.1% [20, 21]. From the 1922 participants, 1458 were of employable age at the time of the study, defined as the age range from 16 to the Swiss statutory retirement age of 65 for men and 64 for women. The response rate in the employable age group was 61.9%.

### Measures

**Fig 1:**
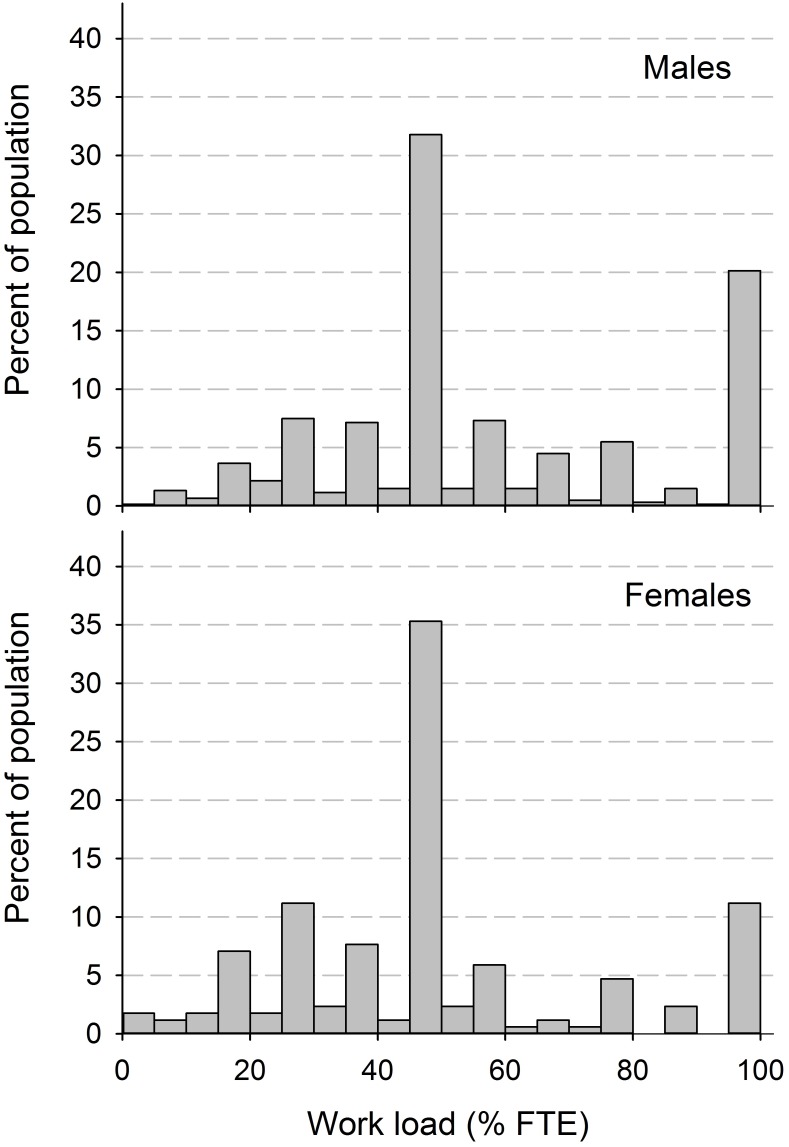
Distribution of amount of work performed for males and females as percent of full-time equivalent in the general population.

The primary outcome of the study was self-reported LMP, defined as employment or self-employment at the time of the survey for at least one hour/week and coded as a dichotomous variable (0 = no LMP; 1 = LMP) [22, 23]. This definition included competitive, sheltered and supported employment. The secondary outcome was the amount of work performed, defined as full-time equivalent (FTE) in percent of the reference level of 42 hours per week in the general Swiss population for those with LMP. To capture the clustered, multimodal distribution in work load (Fig 1) and to facilitate regression modelling, amount of work was recoded as a ordinal categorical variable with the following levels: ≤30%, 31%-60%, 61%-90%, and >90% (full-time).

Independent variables comprised age at the time of the survey in years, gender, years of formal education according to the International Standard Classification of Education combining school and vocational training [24], lesion level (paraplegia vs. tetraplegia), type of lesion (complete vs. incomplete), etiology (traumatic vs. non-traumatic), and time since SCI in years. All independent variables were also derived from self-report. Previous analyses showed that, with the exception of education, all self-reported demographic and lesion characteristics were in substantial agreement with medical record data [20].

General population data for the comparison of employment rates were obtained from the Swiss Federal Statistical Office and represent Swiss labor market statistics for the SwiSCI data collection period (third quarter of 2011) [25].

### Data analysis

#### Basic principles and descriptive analyses

Statistical analyses were performed using the packages Stata version 13.1 (StataCorp, Texas, USA) and R version 3.1.3 (The R Foundation for Statistical Computing). Reporting of methodology and results follows the STROBE guidelines [26].

Descriptive statistics of study participants’ characteristics were provided for the overall sample as well as stratified by employment status. Unless indicated otherwise, descriptive analysis was based on crude data, not accounting for missing data that resulted from either unit-nonresponse (nonparticipation in the survey) or item-nonresponse (nonexistence of data on a specific survey item). Regression analyses of the primary and secondary outcomes were adjusted for both unit and item nonresponse. Unless reported otherwise, results of adjusted analysis were similar to those of unadjusted analysis.

To account for unit-nonresponse, we used sampling weights which were defined as the inverse of the propensity score of participation in the survey [27, 28]. Propensity scores were derived from a multivariable nonresponse analysis that indicated limited nonresponse bias in relation to membership of the SPA, current age, and time since SCI [20]. Item nonresponse, i.e. missing values in explanatory variables or outcomes, was handled using a random forest imputation technique (R-package missForest [29]).

#### Primary outcome: employment status

The primary outcome employment status (employed or self-employed vs. not employed at time of the study) was analyzed with logistic regression models using the following predictor variables: current age (years), gender (reference: female), attained formal education (years), level of lesion (reference: paraplegia), type of lesion (reference: incomplete), etiology (reference: non-traumatic), and time since SCI (years). Predictor variables were used in univariable and multivariable analysis to derive unadjusted and mutually adjusted odds ratios with corresponding 95% confidence intervals (CI), respectively. To evaluate potential effect modification with regards to lesion characteristics, an interaction term between lesion level and lesion type was evaluated separately in the multivariable model. To systematically explore potential nonlinear relationships between employment status and the continuous variables age, years of education, and time since SCI, we used a fractional polynomials algorithm with a predefined set of powers (i.e., −2,−1,−0.5, 0, 0.5, 1, 2, 3; with 0 representing the logarithmic transformation), allowing for repeated powers to further enhance modelling flexibility, and favoring simpler models unless more complicated functions showed superior fit [30–32]. The statistical significance of predictor variables was tested globally (i.e., jointly across all transformations or categorical levels) using adjusted Wald-tests, which adequately account for the usage of robust standard errors in presence of sampling weights. Marginal predictions of employment rates with 95% CI were also derived for graphical display of the relationship of employment rates with continuous predictor variables.

To compare employment rates in SCI with the general population, we used Poisson regression with an offset term to derive age- and gender-standardized employment rate estimates [33, 34]. Count data comprised the number of employed people in the SCI sample stratified by age group (16–24, 25–39, 40–54, 55–64 years of age), gender, and lesion level. The offset term (i.e., the fixed part of the linear predictor) consisted of the log of the expected counts that were calculated by multiplying the number of people in the respective 16 strata with the corresponding age- and gender-specific employment rate in the general population. The average of the individual sampling weights was used as stratum-specific sampling weights. We thus derived employment rate ratios and subsequently rate differences (both with 95% CI) that were standardized to the general population. Modeling assumptions were tested using the deviance and Pearson Goodness-of-Fit (GOF) tests. To gain insight as to whether age and gender differences in employment rates are specific to the SCI population, we also compared effect sizes from models with and without an offset.

#### Secondary outcome: amount of work performed

We used stereotype logistic regression [35–37] to evaluate associations of ordinal levels of the amount of work performed with demographic and injury-related characteristics. Brant testing following initial ordinal logistic regression [38] indicated that the proportional odds assumption was not met (*χ*^2^ (14) = 42.83, p < 0.001). Stereotype logistic regression provides an alternative method that seeks to minimize the number of parameters, while preserving an adequate model fit by enforcing ordering constraints on adjacent ordinal levels that are statistically indistinguishable. We used the lowest FTE category (≤30%) as base level (reference) and imposed a corner constraint on the highest FTE category (>90%) to make the model identifiable. The distinguishability of adjacent ordinal levels (excluding the base level) was evaluated using Wald-test. From the final multivariable model we derived adjusted odds ratios for being in a given FTE category as compared to being in the lowest FTE category for all predictor variables. Global significance testing using Wald-tests was applied to derive p-values in multivariable modeling. Marginal predictions at means were also derived as to graphically illustrate the predicted distribution of participants over the ordinal levels of FTE in persons with SCI participating in the labor market.

The amount of work (FTE) performed by people with SCI was compared to the general population using basic descriptive statistics that contrasted the distribution in SCI with the average FTE in the general population. Subsequently, the percentage of persons with SCI working less, the same or more than their respective age and gender group in the general population was analyzed using Wilcoxon signed-rank test.

## Results

### Employment rates

#### Person characteristics and employment rates in SCI

Study participants’ demographic and injury characteristics are given in Table 1. The majority of the participants was middle-aged, male, had high levels of education, paraplegia, and a traumatic etiology. On average around 53% were employed at the time of the study. Weighted employment rates were only slightly different from crude rates.

**Table 1:** Characteristics of study participants for the total study population and stratified by employment status.

Parameter [n missing]	Total		Not employed		Employed		Employed %
Indicator variables	N	%	n	%	n	%	(Weighted estimate)^a^
Total	1458	100	674	46.6	771	53.4	53.4
Gender [0]							
Male	1067	73.2	454	43.0	601	57.0	57.0
Female	391	26.8	220	56.4	170	43.6	43.8
Age (years) [0]							
16–24	65	4.5	38	58.5	27	41.5	42.4
25–39	330	22.6	125	38.2	202	61.8	62.0
40–54	645	44.2	271	42.3	370	57.7	57.3
55-63/64^#^	418	28.7	240	58.3	172	41.7	41.5
Education level [30]							
Compulsory (≤ 9 years)	123	8.6	89	73.6	32	26.4	25.7
Vocational (10–12 years)	346	24.2	203	59.2	140	40.8	40.9
Secondary (13–16 years)	686	48.0	287	41.8	399	58.2	58.4
University (≥ 17 years)	273	19.1	80	29.3	193	70.7	70.7
Lesion level [17]							
Paraplegia	990	68.7	424	43.0	563	57.0	56.7
Tetraplegia	451	31.3	244	54.5	204	45.5	46.4
Lesion type [13]							
Incomplete	793	54.9	381	48.4	407	51.6	52.1
Complete	652	45.1	288	44.2	363	55.8	55.4
Time since injury (years) [28]							
< 1	19	1.3	11	57.9	8	42.1	43.1
1–5	251	17.5	128	51.8	119	48.2	48.2
6–10	262	18.3	114	43.8	146	56.2	55.7
11–15	217	15.2	105	48.4	112	51.6	51.5
16–20	171	12.0	70	40.9	101	59.1	59.2
21–25	176	12.3	84	47.7	92	52.3	52.6
26–30	136	9.5	57	41.9	79	58.1	57.8
31–35	81	5.7	38	46.9	43	53.1	53.3
36+	117	8.2	54	46.1	63	53.9	53.9
Etiology [17]							
Traumatic	1206	83.7	533	44.4	668	55.6	55.8
Non-traumatic	235	16.3	137	58.5	97	41.5	41.3
Continuous variables	Median	(IQR)	Median	(IQR)	Median	(IQR)	Median (IQR)
Age	48	(39–55)	49	(40–58)	46	(38–53)	46 (38–53)
Years of education	13	(12–16)	13	(11–14)	14	(13–17)	14 (13–17)
Years since SCI	14	(7–24)	13	(6–24)	15	(7–25)	15 (7–23)

Notes: Percentage were calculated using non-imputed data, by employment status ignoring 13 cases with missing employment status.

^a^ Weighted for unit non-response.

^#^ 63 in females, 64 in males.

#### Predictors of employment status in SCI

The unadjusted analyses of employment status revealed clear associations with gender, age, years of formal education, lesion level and etiology, but not with lesion type and time since SCI. Most of these associations were also found in the adjusted, multivariable analysis with the exception of time since SCI and etiology, which showed slightly higher and lower odds ratios, respectively, than in the univariable analysis (Table 2). Further, there was no indication for an interaction between type and level of lesion in the multivariable model (*χ*2 (1) = 0.06, p = 0.81). Adjusted odds for being employed or self-employed were 73% higher in males than in females and 78% higher in persons with paraplegia as compared to tetraplegia. In addition, the adjusted odds were elevated by 13% per ten-year increase in time since SCI, while varying non-linearly with age and years of education.

**Table 2:** Predictors of employment status.

Parameter	Univariable analysis		Multivariable analysis	
	Unadjusted odds ratio (95% CI)	P-value^#^	Adjusted odds ratio (95% CI)	P-value^#^
Gender		< 0.0001		< 0.001
Female	1.00		1.00	
Male	1.66 (1.31–2.10)		1.73 (1.33–2.25)	
Age (/10 years)		< 0.0001		< 0.0001
Age^3	1.05 (1.02–1.08)		1.05 (1.02–1.08)	
Age^3 * ln(Age)	0.97 (0.96–0.99)		0.97 (0.96–0.99)	
Education (/10 years)		< 0.0001		< 0.0001
Education^2	14.27 (6.53–31.16)		12.03 (5.42–26.70)	
Education^3	0.42 (0.30–0.57)		0.44 (0.32–0.62)	
Lesion level		< 0.001		< 0.0001
Tetraplegia	1.00		1.00	
Paraplegia	1.53 (1.22–1.91)		1.78 (1.40–2.27)	
Lesion type		0.18		0.24
Complete	1.00		1.00	
Incomplete	0.87 (0.70–1.07)		1.15 (0.91–1.47)	
Time since injury		0.21		0.027
(/10 years)	1.06 (0.97–1.16)		1.13 (1.01–1.27)	
Etiology		< 0.0001		0.083
Non-traumatic	1.00		1.00	
Traumatic	1.76 (1.32–2.35)		1.35 (0.96–1.91)	

Notes: Given are odds ratios derived from logistic regression analysis with being employed or self-employed as dependent variable.

^#^ From Wald test (following weighted logistic regression analysis with robust standard errors).

All analyses were adjusted for both unit and item nonresponse.

**Fig 2:**
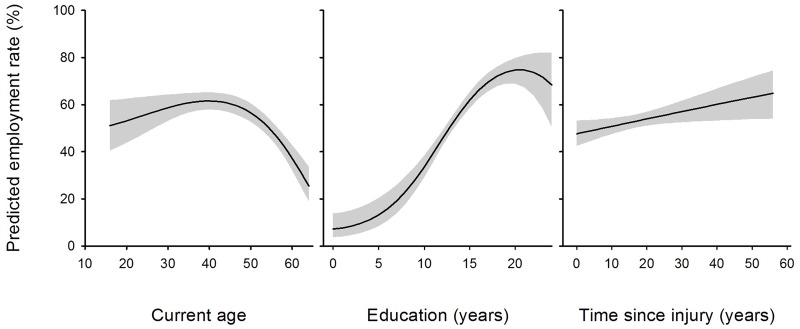
Adjusted marginal predictions of employment rates for continuous predictor variables in Table 2. Predictions are at means for all other variables in the multivariable model with grey areas indicating the 95% confidence interval.

Fig 2 depicts the associations for the continuous variables as marginal predictions of employment rates. Employment rates showed an asymmetrical concave relationship with age peaking at about 60% at around age 40, and an s-shaped relationship with years of education, gradually increasing from low rates to about 45% at around 12 years (vocational) and then gradually leveling off at 70% to 75% at 17 or more years (university level) of formal education. The study population further showed an about 3.2% increase in LMP with each ten years since SCI. Marginal predictions (95% CI) for categorical variables in Table 2 from the adjusted model were: 56.5% (53.2–59.8) and 42.9% (37.6–48.4) for males and females; 57.3% (53.9–60.7) and 43.0% (38.3–47.9) for the paraplegic and tetraplegic group; 54.5% (50.6–58.3) and 50.9% (46.5–55.3) for the incomplete and complete lesion group; and 54.1% (51.0–57.1) and 46.5% (38.8–54.4) for persons with traumatic SCI as compared to non-traumatic SCI.

#### Employment rates of people with SCI in comparison with the general population

The Poisson regression model for evaluating standardized employment rate ratios demonstrated good statistical fit and revealed associations with gender, age group and lesion level (Table 3).

**Table 3:** Adjusted employment rate ratios for strata that are standardized to age- and gender-specific employment rates of the general population.

Parameter	Standardized LMP rate ratio (95% CI)	P-value
Gender		0.049
Female	1.00	
Male	1.13 (1.00–1.28)	
Age group		0.012
16–24	1.19 (0.87–1.62)	
35–39	1.28 (1.10–1.47)	
40–54	1.18 (1.03–1.35)	
55-63/64^#^	1.00	
Lesion level		< 0.001
Tetraplegia	1.00	
Paraplegia	1.26 (1.12–1.41)	

Notes: Stratified estimates with 95% CI were derived from a multivariable Poisson regression model with expected age- and gender-specific employment rate, log-transformed, as offset. Goodness-Of-Fit (GOF) testing confirmed that modelling assumptions were fulfilled (deviance GOF, *χ*2(10) = 8.97, p = 0.53; Pearson GOF, *χ*2(10) = 9.51, p = 0.48).

^#^ 63 in females, 64 in males

**Fig 3:**
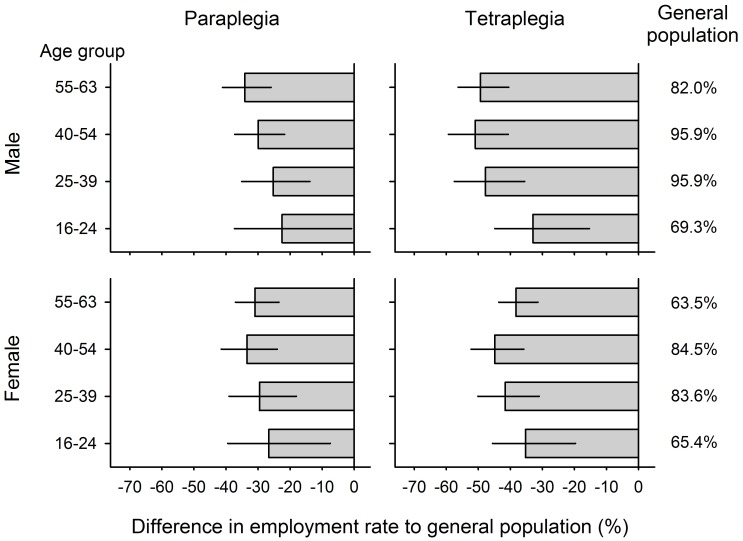
Age- and gender-standardized difference in employment rates between persons with SCI and the general population. For stratified estimates of overall employment rate in paraplegia and tetraplegia, see Table 1.

Adjusted employment rates were estimated as 1.13 times higher in males than in females (a 13% increase); between 1.18 and 1.28 times higher in the persons from the three youngest age groups as compared to the oldest age group (elevations between 18% and 28%); and 1.26 times higher in paraplegia as compared to tetraplegia (a 26% increase). Fig 3 shows the estimated differences of employment rates in people with SCI as compared to the general population, by gender, age and lesion level. On average, the employment rate of people with SCI was around 30% lower than in the general population. Males with tetraplegia aged between 40 and 54 showed the greatest difference, while men with paraplegia aged between 16 and 24 years showed the smallest difference in comparison to the general population.

### Amount of work performed as FTE

#### Descriptive analysis of work load in SCI

From the 771 employed persons in our sample, the vast majority (81.7%) worked part-time (here defined as less than 91% of FTE) with a median of 50% FTE (IRQ: 40%-80%). In total and across all groups, the majority of participants reported 31–60% FTE. Part-time work was particularly more frequent in females, older employees and persons with lower education. Regarding lesion characteristics, persons with complete lesions or having a traumatic etiology less commonly worked full-time (Table 4).

**Table 4:** Descriptive statistics of amount of work performed (in % FTE) for employed persons (total n = 771).

Parameter	Work load; n (%)			
	≤30%	31%-60%	61%-90%	>90%
Total	134 (17.4)	394 (51.1)	102 (13.2)	141 (18.3)
Gender				
Male	92 (15.3)	301 (50.1)	86 (14.3)	122 (20.3)
Female	42 (24.7)	93 (54.7)	16 (9.4)	19 (11.2)
Age (years)				
16–24	2 (7.4)	9 (33.3)	7 (25.9)	9 (33.3)
25–39	18 (8.9)	109 (54.0)	28 (13.9)	47 (23.3)
40–54	68 (18.4)	194 (52.4)	51 (13.8)	57 (15.4)
55-63/64	46 (26.7)	82 (47.7)	16 (9.3)	28 (16.3)
Education level				
Compulsory schooling	7 (21.9)	21 (65.6)	3 (9.4)	1 (3.1)
Vocational training	30 (21.4)	72 (51.4)	16 (11.4)	22 (15.7)
Secondary education	68 (16.8)	215 (53.0)	50 (12.3)	73 (18.0)
University education	29 (15.0)	86 (44.6)	33 (17.1)	45 (23.3)
Lesion level				
Paraplegia	87 (15.3)	298 (52.6)	76 (13.4)	106 (18.7)
Tetraplegia	47 (23.0)	96 (47.1)	26 (12.8)	35 (17.2)
Lesion type				
Incomplete	65 (16.0)	170 (41.8)	66 (16.2)	106 (26.0)
Complete	69 (19.0)	224 (61.5)	36 (9.9)	35 (9.6)
Time since injury (years)				
≤ 5	23 (18.1)	56 (44.1)	29 (22.8)	19 (15.0)
6–15	44 (16.7)	117 (44.5)	37 (14.1)	65 (24.7)
16–25	33 (17.0)	110 (56.7)	21 (10.8)	30 (15.5)
≥ 26	34 (18.2)	111 (59.4)	15 (8.0)	27 (14.4)
Etiology				
Traumatic	118 (17.5)	358 (53.1)	84 (12.5)	114 (16.9)
Non-traumatic	16 (16.5)	36 (37.1)	18 (18.6)	27 (27.8)

#### Predictors of amount of work performed by people with SCI

**Fig 4:**
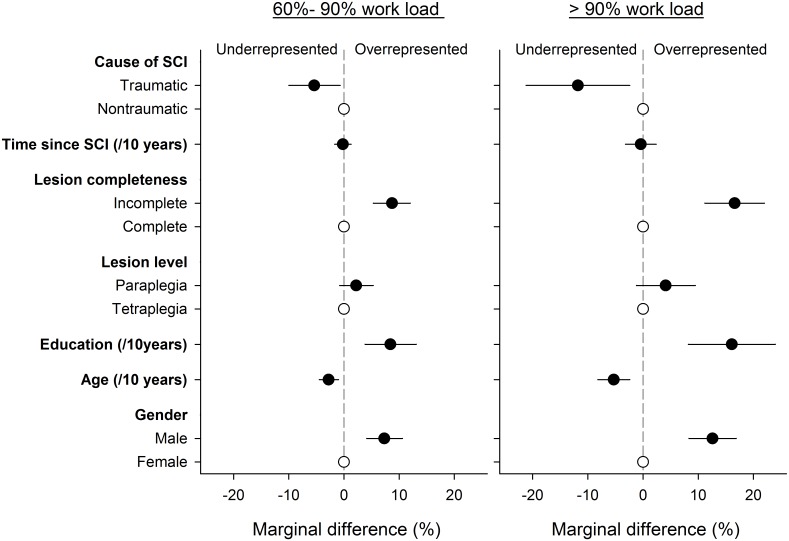
Marginal prediction of the relative distribution of participants over the ordinal levels of the amount of work performed (FTE, %) in relation to sociodemographic and lesion characteristics. Display is restricted to the two highest level categories with 60%-90% or >90% FTE noting that the two lowest categories predictably showed opposite marginal differences, essentially producing a balancing mirror image. Estimates are at mean values for other parameters using the stereotype logistic regression model in Table 3. Open symbols indicate the reference category for each parameter; solid symbols and error bars the percent difference with 95% confidence interval for other parameter classes as well as for continuous variables. Reading example: Given the overall gender distribution, men are overrepresented by 12.6% (95% CI: 8.3%-16.9%) in the >90% work load category.

Stereotype logistic regression with lowest (≤30%) FTE group as base (reference) category and highest (>90%) as constraint (identification) category was fitted. Tests indicated that other levels were distinguishable, implying that the number of ordinal levels in the model could not be further constrained without decrease in model fit. Distinguishability statistics for adjacent levels of FTE were as follows: comparing “>90%” to”61%-90%”, adjusted Wald-test, F(1, 770) = 4.37, P = 0.037; and”61%-90%” to “31%-60%”, adjusted Wald-test, F(1, 770) = 20.22, P < 0.001. Table 5 gives adjusted odds ratios for relevant patient characteristics for being in a given FTE category with reference to the lowest FTE category (i.e., ≤30%). Men, those with younger age and higher education showed significantly increased odds of working more. In addition, persons with an incomplete lesion reported gradually higher FTE as compared to those with a complete lesion, while those with a traumatic lesion worked less than people with a non-traumatic lesion. Time since SCI did not systematically affect the amount of work performed. For a visualization of the marginal predictions of person distributions over FTE categories see Fig 4.

**Table 5:** Predictors of amount of work performed in % FTE in employed persons.

		FTE, %		
Parameter	P-value^#^	31%-60%	61%-90%	>90%
Gender	< 0.0001			
Female		1.00	1.00	1.00
Male		1.26 (0.69–2.30)	2.99 (1.45–6.17)	4.15 (2.09–8.21)
Current age (/10 years)	0.0034	0.92 (0.55–1.55)	0.68 (0.46–1.03)	0.61 (0.44–0.85)
Education (/10 years)	< 0.0001	1.28 (0.62–2.67)	3.21 (1.41–7.32)	4.54 (2.11–9.78)
Lesion level	0.17			
Tetraplegia		1.00	1.00	1.00
Paraplegia		1.07 (0.65–1.75)	1.37 (0.76–2.47)	1.50 (0.84–2.69)
Lesion type	< 0.0001			
Complete		1.00	1.00	1.00
Incomplete		1.31 (0.78–2.18)	3.49 (1.87–6.52)	5.06 (3.02–8.49)
Time since injury (/10 years)	0.80	0.99 (0.78–1.27)	0.97 (0.69–1.37)	0.97 (0.75–1.25)
Etiology	0.0061			
Non-traumatic		1.00	1.00	1.00
Traumatic		0.85 (0.40–1.82)	0.47 (0.23–1.00)	0.38 (0.19–0.76)

Notes: Adjusted odd ratios (95% CI) are from stereotype logistic regression with category of ≤30% work load as reference.

^#^ P-values from adjusted Wald-test

#### Comparison of work load with general population

The crude median FTE (IQR) in participants who worked was 50% (40%-80%), with higher amounts of work performed by males (50%; 47%-80%) as compared to females (50%; 34%-60%). The majority of employed men and women with SCI worked less than the general population. Further stratification by age group and lesion level (Table 6) revealed a tendency for reduced work in males with older age. Correspondingly, an elevated proportion of persons reported to work less than the general population, ranging from 69.4% to 86.5% across the middle and upper age groups. Females of the intermediate age groups reported similarly high frequencies of reduced work, while trends were indistinct for the youngest and oldest age group due to limited sample size.

**Table 6:** Descriptive statistics of the amount of work performed (FTE, %) by gender, age group and lesion level in comparison with the general population.

Gender, age group	General population mean (GPM)	Paraplegia				Tetraplegia			
		N	Median (IQR)	Proportion below GPM	P-value	N	Median (IQR)	Proportion below GPM	P-value
Male									
16–24	61.7	15	80 (60–100)	33.3	0.019	6	70 (50–100)	50.0	0.46
25–39	94.1	108	60 (50–100)	69.4	< 0.0001	38	50 (40–80)	81.6	< 0.0001
40–54	97.3	208	50 (50–70)	85.1	< 0.0001	81	50 (40–80)	80.2	< 0.0001
55–64	80.6	108	50 (39–80)	77.8	< 0.0001	37	50 (30–50)	86.5	< 0.0001
Female									
16–24	53.4	3	-	-	0.10	3	-	-	0.59
25–39	62.2	40	50 (50–60)	80.0	0.015	16	50 (33–52)	81.3	0.13
40–54	60.6	62	50 (30–60)	80.6	< 0.001	19	40 (25–80)	68.4	0.11
55–63	42.7	23	50 (30–50)	47.8	0.46	4	43 (33–70)	50.0	1.00

Notes: P-values are from a Wilcoxon signed-rank test.

Abbreviations: IQR: Interquartile range.

## Discussion

This study analyzed LMP of persons with SCI living in Switzerland in terms of employment rates and the amount of work performed. About half of the study population participated in the labor market, to a great extent in part-time employment. Employment rates were markedly reduced as compared to the general population. While females with complete tetraplegia had the lowest employment rates in absolute terms, older males with tetraplegia had the greatest disadvantage as compared to men from the general population. Employed men with tetraplegia aged 55 and above also showed the most accentuated reduction in work hours in terms of FTE as compared to the general population.

The found employment rate of 53.4% is around 10% lower than that reported by Marti et al. in 2010 [18]. This is likely due to self-selection bias in the latter study which was flagged as a LMP survey. Employed people may have found this topic more interesting or relevant to their lives than unemployed people. By contrast, the SwiSCI community survey was a survey on functioning in SCI in general, reducing the probability of self-selection due to employment status.

The observed employment rates for Switzerland are comparable to those from Scandinavian countries and the Netherlands averaging around 50% [16, 39], whereas LMP is significantly lower in Southern Europe and globally (37%) [5]. Employment rates of persons with SCI considerably vary by country and continent and range from 11.5% to 74.0% [16], with highest rates in Europe (51%) and lowest in North America (30%) [40] However, the interpretation of these figures is difficult as most studies do not include persons with non-traumatic SCI and use different definitions of employment (e.g., not exclusively paid employment). Reported employment rates may also be considerably affected by the time period under consideration and the sampling frame of respective studies. Also, cited studies do not consider age and gender adjusted risk differences as compared to the general population. It is therefore highly recommended that upcoming between-country comparisons include general population estimates of employment rates in order to determine the relative disadvantage of the SCI population.

Demographic and injury-related factors associated with LMP largely confirm previous research [13, 17]. Our finding that age was non-linearly related with LMP suggests a gradual increase of employment rates between the age of 16 and 40 and then an increasing decline. The later decline might be partially mediated by having pain, which along with increasing age reduced the likelihood of LMP in subsample of participants who reported on their health conditions in a subsequent survey module [41]. Regarding years of formal education, our findings suggest that people with SCI in Switzerland benefit most from an increase between 7 and 15 years of formal education. As education is a modifiable factor, respective interventions are advised. Future research is needed though to compare trajectories with those of the general population, with other countries as well as between different sub-groups of the SCI population. The result of a linear increase in LMP with time since SCI (when adjusted for age) suggests an adjustment process after SCI, potentially involving physical and psychosocial recovery as well as re-training or completion of education in some cases [42]. It is remarkable that this linear relationship continues after the first 5–10 years after SCI.

The literature on the amount of work performed after SCI is sparse and cut-offs to define full-time employment differ, thus limiting comparability. In contrast to our findings indicating a high prevalence of part-time employment, some studies indicated rates of 70.3% [43] and 80.0% [44] full-time employment among the working SCI population. However, methodological issues seem to explain the large differences compared to our findings, as full-time employment was defined as working more than 30 hours a week [44] or was not defined at all [43]. In general, data seem to indicate a shift towards reduced work load after SCI [43], potentially due to physical capacity as well as increased amounts of time needed for other activities. A recent study from Van der Meer and colleagues estimated a median of 13 hours extra time per week needed for self-care, bowel-bladder care, transportation, and handicap-management [45]. Our finding of a higher work load in men mainly reflects patterns in the Swiss general population, while an increased work load of people with incomplete lesions may be related to physical capacity. In line with our findings, higher education was also associated with an increase in the time worked [44], which could be in part explained by less physically demanding jobs. Interestingly, while we found a trend for a lower employment rate of people with non-traumatic SCI, those employed tended to work more, a finding that may be due to differential economic needs which in turn might be mediated by different social insurance benefits depending on whether SCI was due to accident or illness.

Our study has several limitations that warrant discussion. Our study sample may not be representative of the target population. However, we minimized this risk by recruiting participants using a combination of several databases, including rehabilitation hospitals, the Swiss patient association and a home-care institution for SCI [20]. Given our sampling frame, we can also not exclude unit-nonresponse bias related to LMP, because information regarding LMP status was not available at the time of survey invitation and was thus not accounted for in the sampling weights [20]. The cross-sectional data examined further preclude the study of individual trajectories related to age and cohort. Most importantly, other factors than demographics and injury-related variables, such as differences in the environment or psychological resources that have not been accounted for in this study, may explain variation in LMP. In order to identify suitable intervention targets for an enhanced LMP of persons with SCI, future empirical and experimental research needs to address modifiable environmental and personal factors in relation to bodily capacity [15, 17, 46–49]. In the comparison of the amount of work performed by employed people from the SCI and the general population, stratification by gender, age group and lesion level yielded small sample sizes in some cases precluding meaningful interpretation.

Several strengths of our study warrant mentioning as well. We used a large sample of people with SCI based on the combination of several databases. As we had basic demographic information for the sampled population, we were able to correct for unit-nonresponse by employing inverse probability weights. Moreover, we had access to data about LMP of the general population from the Swiss Federal Statistical Office and could therefore provide a detailed comparison between people with SCI and the general population. Furthermore, our analysis considered the possibility of non-linear relationships between LMP and predictors.

## Conclusions

LMP of persons with SCI is comparatively high in Switzerland. LMP after SCI is, however, considerably lower than in the general population. Future research needs to show whether the reduced LMP in SCI reflects individual capacity adjustment, contextual constraints on higher LMP, or both.
